# FinFET 6T-SRAM All-Digital Compute-in-Memory for Artificial Intelligence Applications: An Overview and Analysis

**DOI:** 10.3390/mi14081535

**Published:** 2023-07-31

**Authors:** Waqas Gul, Maitham Shams, Dhamin Al-Khalili

**Affiliations:** Department of Electronics, Carleton University, 1125 Colonel Bay Drive, Ottawa, ON K1S 5B6, Canada

**Keywords:** FinFET-SRAM, SRAM cell, compute-in-memory, CIM accelerators, convolution neural network (CNN), multiply and accumulate (MAC)

## Abstract

Artificial intelligence (AI) has revolutionized present-day life through automation and independent decision-making capabilities. For AI hardware implementations, the 6T-SRAM cell is a suitable candidate due to its performance edge over its counterparts. However, modern AI hardware such as neural networks (NNs) access off-chip data quite often, degrading the overall system performance. Compute-in-memory (CIM) reduces off-chip data access transactions. One CIM approach is based on the mixed-signal domain, but it suffers from limited bit precision and signal margin issues. An alternate emerging approach uses the all-digital signal domain that provides better signal margins and bit precision; however, it will be at the expense of hardware overhead. We have analyzed digital signal domain CIM silicon-verified 6T-SRAM CIM solutions, after classifying them as SRAM-based accelerators, i.e., near-memory computing (NMC), and custom SRAM-based CIM, i.e., in-memory-computing (IMC). We have focused on multiply and accumulate (MAC) as the most frequent operation in convolution neural networks (CNNs) and compared state-of-the-art implementations. Neural networks with low weight precision, i.e., <12b, show lower accuracy but higher power efficiency. An input precision of 8b achieves implementation requirements. The maximum performance reported is 7.49 TOPS at 330 MHz, while custom SRAM-based performance has shown a maximum of 5.6 GOPS at 100 MHz. The second part of this article analyzes the FinFET 6T-SRAM as one of the critical components in determining overall performance of an AI computing system. We have investigated the FinFET 6T-SRAM cell performance and limitations as dictated by the FinFET technology-specific parameters, such as sizing, threshold voltage (V_th_), supply voltage (V_DD_), and process and environmental variations. The HD FinFET 6T-SRAM cell shows 32% lower read access time and 1.09 times better leakage power as compared with the HC cell configuration. The minimum achievable supply voltage is 600 mV without utilization of any read- or write-assist scheme for all cell configurations, while temperature variations show noise margin deviation of up to 22% of the nominal values.

## 1. Introduction

Modern artificial intelligence (AI) deploys deep neural networks (DNNs) for quick and self-sufficient operations. Data-centric DNNs require huge amounts of data for their training and inference [1,2]. Consequently, data traffic between memory and processing units has increased immensely and choked overall system performance. Researchers have proposed the compute-in-memory (CIM) concept as a solution to overcome data movement bottlenecks to ensure AI platform performance.

Modern edge computing devices are now generating data on the order of the tera-bits per second. Huge data inference in general processing units demands extended amounts of time for the on and off chip movement and extra computational resources. Thus, CIM addresses these challenges by reducing off-chip traffic and data movement for cloud computing.

The CIM approach is accomplished either based on the mixed-signal or all-digital signal domains. The mixed-signal approach evolved earlier, as it achieves better energy efficiency [3]. However, the analog signal nature restricts such CIM to fewer bits precision and needs additional peripheral circuitry [4]. Hence, this approach is not feasible for the accuracy of critical AI systems. Howbeit, the digital signal domain approach does not suffer from accuracy or bit-precision issues, but it uses dedicated on-chip circuitry for arithmetic operations, known as near-memory computing (NMC), and pushes hardware and power overhead high. Recently, modified SRAM cells achieved better efficiency by performing some computations inside the cell, known as in-memory computation (IMC), and the rest of the computations with arithmetic units placed near the memory. For a broad range of AI applications coverage, near-memory and in-memory computations optimize frequent NN operations such as MAC. The all-digital signal domain SRAM CIM [5] is more accurate and performance-centric because of its digital signal domain operations and adaptability to the advanced technological nodes.

**Figure 1:**
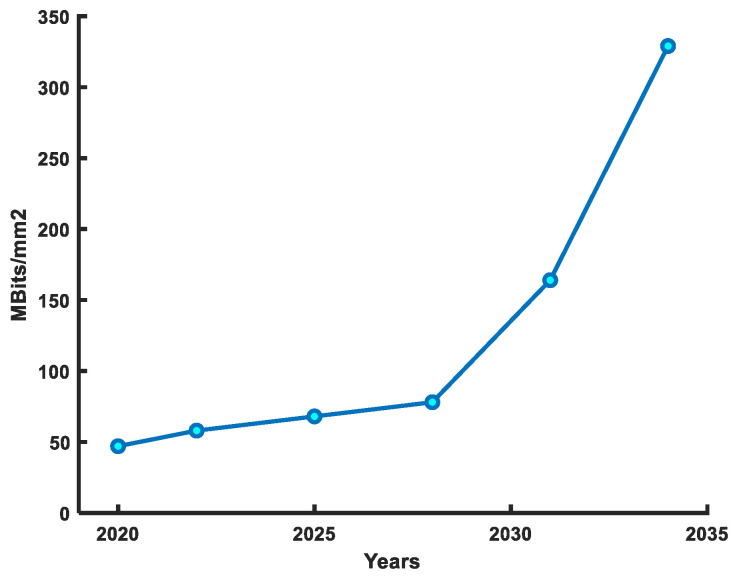
SRAM density improvement roadmap as per IRDS 2022 [10] guidelines.

6T-SRAM cell performance has improved with the evolution of the transistor. Over the last two decades, the 6T-SRAM cell has evolved from planar transistor to the 3D-FinFET structure to improve design density, power, and performance. Figure 1 shows the SRAM density trends and projections. In modern technological nodes, the 6T-SRAM cell suffers from many challenges, such as low-voltage operation reliability, leakage current, soft errors, security-aware design requirements, and half-select problems [6,7,8,9]. The severity of these issues has increased in the FinFET 6T-SRAM cells. Because in modern technological nodes low supply voltage usage saves power but poses a threat to cell stability, leakage current has become comparable to the on-current; thus, leakage power is a now significant portion of total power consumption, and decreased geometrical dimensions make it easier to alter internal node value, which increases soft error probability. To discourage the side-channel attacks to steal data from the cell, FinFET 6T-SRAM needs design alterations at the cell level. Furthermore, in a higher SRAM density design, only row or column selected cells, known as half-select, can flip their values. Therefore, the FinFET 6T-SRAM cell design needs a comprehensive analysis to ensure a reliable operation in CIM solutions for AI applications.

The rest of the article is organized as follows. Section 2 gives background knowledge about DNN structures and the 6T-SRAM cell’s basic operation. Section 3 presents silicon-verified CIM digital accelerators (NMC) and custom-designed CIM SRAMs (IMC) at the system-on-chip (SoC) level. Next, Section 4 provides a comprehensive analysis of challenges to the modern FinFET 6T-SRAM cell as a direct impact of transistor scaling. Finally, Section 5 concludes this paper.

## 2. Background

This section provides background knowledge about NN structures and the 6T-SRAM cell as the foundation for the FinFET 6T-SRAM CIM comprehension.

### 2.1. Deep Neural Networks (DNNs)

**Figure 2:**
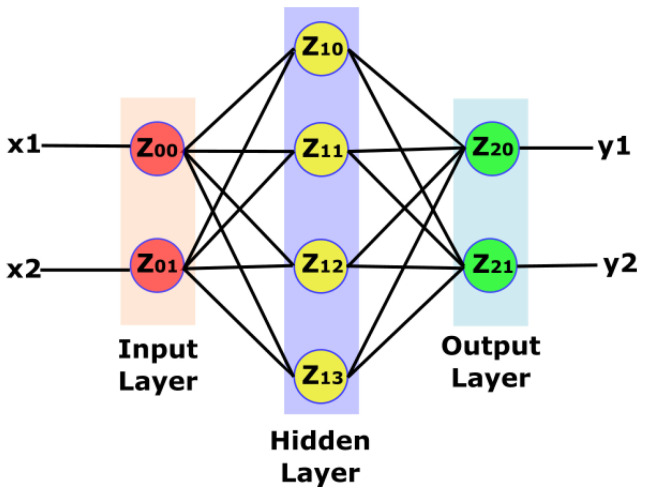
Neural network structure: input, output and one hidden layer.

In Figure 2, we see a three-layer neural network. The first layer, known as the input layer, uses input values. The second layer or hidden layer is composed of hidden neurons (z) whose activation is dependent on the synapses’ values and a specific function, known as the activation function. The third layer is referred to as the output layer. Each layer connects itself to the next layer’s neurons via connections or synapses. These connections carry weights; an NN trainer tunes weight values during the training phase.

Moreover, the number of hidden layers and the number of neurons in each layer are not fixed. Variation in hidden layers and neuron number is a tradeoff between NN complexity and accuracy. Therefore, these two numbers must be chosen to achieve acceptable accuracy, while keeping the complexity level low. Each weight multiplies itself with the output value from the previous layer neuron. Afterward, each neuron accumulates multiplication results from all synapses and applies an activation function on multiply accumulated value. A neuron usually uses sigmoid, rectified-linear, tangent, or binary functions for activation purposes.

### 2.2. 6T-SRAM Cell

**Figure 3:**
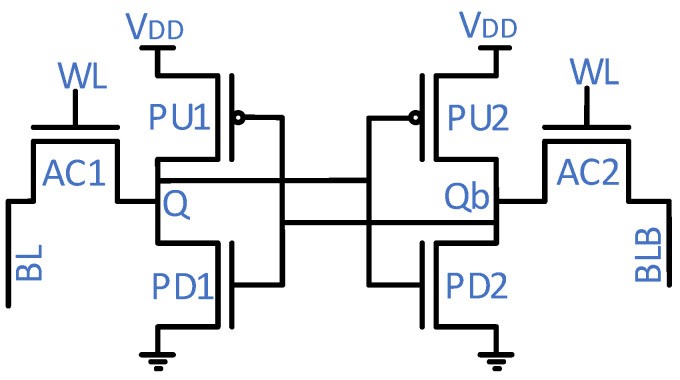
Conventional 6T-SRAM cell structure.

Figure 3 shows a conventional 6T-SRAM cell; it consists of six transistors: pull-up (PU), pull-down (PD), and access (AC). The 6T-SRAM cell operates in either of three modes: hold, read, and write. In the hold mode, both access transistors are off, hence the cell retains the internal nodes’ values. In the read operation, the access transistors activation through the wordline (WL) connects the internal nodes (Q and Qb) to the precharged bitlines (BLs). Then, the SRAM cell puts stored values onto the BLs. The write operation is the inverse of the read operation, i.e., a write driver through the BLs puts value onto the internal nodes. The 6T-SRAM cell transistors’ strength differs to ensure a successful operation. The PD transistors should be the strongest ones, while the AC and PU transistors come in second and third place, respectively, in the strength hierarchy. The pull ratio (the PU-to-AC transistors strength) and cell ratio (the PD-to-AC transistors strength) adjustment tunes the read and write performance of an SRAM cell.

Noise margins are fundamental indicators for measuring noise tolerance levels. Each operational mode, as described above, has a different noise tolerance. To measure the noise tolerance level during each operational mode, an external source injects noise into the internal nodes (Q and Qb). After a certain noise level, an SRAM cell is flipped. That noise level is known as the noise margin for that particular mode of operation.

An SRAM array periphery includes a row decoder and a column decoder to access a particular SRAM location, sense amplifiers to read the memory, write drivers to write data into the memory, and a controller to synchronize control signals to perform overall operations.

## 3. Compute-in-Memory (CIM)

An increase in data traffic between microprocessor and memory due to DNN inference degrades performance and increases power consumption. To reduce the data traffic, a newer version of memory is introduced with some computational capability, known as CIM. Hence, CIM reduces traffic and consequently optimizes performance and power. Different memories are used for CIM implementation. Table 1 lists the fundamental performance parameters of multiple memories cell. A designer can make an informed decision about using a specific memory cell type for a CIM implementation.

**Figure 4:**
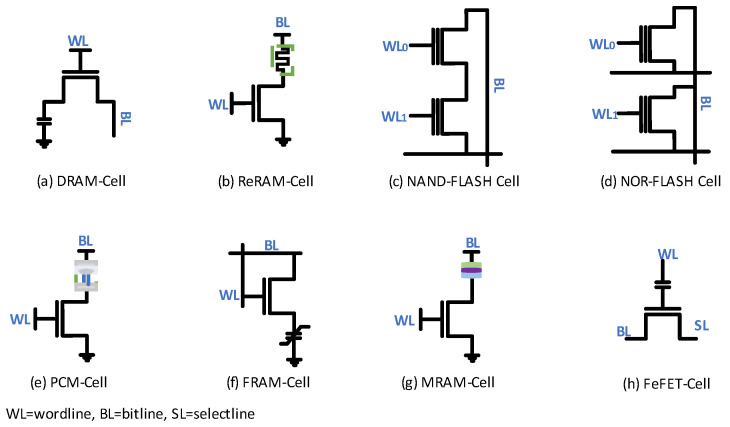
Single-cell configurations of state-of-the-art memories [47,48,49,50,51,52,53,54].

The FinFET 6T-SRAM uses restoring logic but needs six transistors per cell (Figure 3). Due to the restoring logic, retention time of the value stored inside the FinFET 6T-SRAM cell is limited to the duration of supply voltage availability. Figure 4 shows the transistor-level implementation of each memory cell type reported in Table 1, except the FinFET 6T-SRAM cell. Next, dynamic random-access memory (DRAM) suffers from the challenge of leakage current and thus needs a refresher current to keep the charge level maintained. The retention time of a DRAM is in the range of few milliseconds, as it uses a tiny capacitor to store the value. Small storage capacitance keeps retained value duration minimal. Formation of the resistive path in resistive RAM (ReRAM) during write operation pushes the write voltage requirement high, and reliability becomes a challenge in the long run. NAND/NOR flash memories utilize a floating gate transistor for value storage. Both memories, NAND/NOR flash, differ in erase, program, read, reliability, and power consumption. NOR flash accessibility enables its utilization in code execution, while NAND flash has higher density and lower cost compared with the NOR flash counterpart. Phase-change memory (PCM) exploits amorphous and crystalline transitions of phase-change material to store data. However, the high write current and temperature limits PCM endurance. Replacement of dielectric material in DRAM with the ferroelectric material leads to a ferroelectric RAM (FRAM) cell. However, like the DRAM cell, the FRAM cell also needs rewriting after each read value operation. Magneto-resistive RAM (MRAM) unveils magnetic layers as a potential candidate for value storage. The magnetic storage concept makes MRAM performance comparable to DRAM. FRAM reliability, especially under the radiation emission environment, is still a challenge. FeFET uses a ferroelectric layer at the gate to control charge flow between the source and drain. Similar to other nonvolatile memories, this one also suffers from issues of high write voltage.

Besides the memories mentioned in Table 1, some emerging memories are [10] novel magnetic memory, i.e., spin-transfer torque (STT) and spin-orbital torque (SOT), oxide-based resistive RAM (OxRAM), conducting bridging RAM (CBRAM), macromolecular memory, massive storage devices, and MOTT memory. New emerging devices are under research and are still in their infancy stages; therefore, very limited performance data and cell structure details are available.

The 6T-SRAM cell only relies only on transistors (Figure 3). No specific material or properties exploitation, i.e., resistance or magnetization, is needed. Therefore, SRAM cell design keeps pace with technological node scaling. As of today, 3 nm FinFET is the latest technological node, and the FinFET 6T-SRAM manufacturing is also fabricated at that node [11]. Whereas materials-specific transistor structure hinders the rest of the memory cells’ maturity towards modern technological nodes, the restoring logic of the FinFET 6T-SRAM shows better performance (Table 1), and scalability to advanced technological nodes enables it to have lower nominal supply voltage. Dynamic power consumption depends on the square of the supply voltage. Hence, dynamic power consumption is lower for the FinFET 6T-SRAM cell. This makes the FinFET 6T-SRAM cell an appealing choice for CIM AI applications.

### 3.1. SRAM CIM Architectures

**Figure 5:**
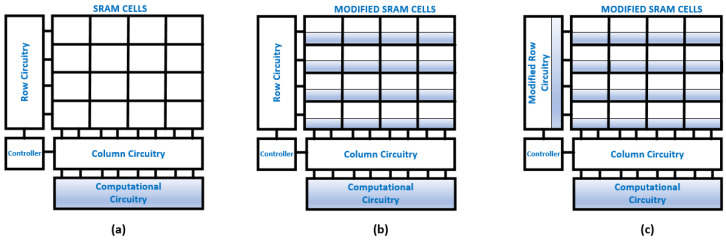
SRAM-based CIM architectures for DNN processing acceleration: (**a**) NMC architecture, (**b**) IMC architecture, and (**c**) customized IMC architecture.

Figure 5 classifies hardware implementations of SRAM-CIM into three architectures. Figure 5a shows the introduction of the additional computational hardware without any other alteration to the memory architecture. In this architecture, the SRAM read operation provides data to the computational unit for NN inference. CIM processors/accelerators adopt this type of hardware using the ASIC implementation approach. The computational unit is composed of standard cell-based arithmetic units and control circuitry for data flow. This requires less time and focuses on data flow/compression efficiency. Therefore, Figure 5a architecture is used in the digital signal domain NMC.

Figure 5b represents modifications in the SRAM cells and extra computational circuits with highlighted blocks to indicate modified cell structures. In this approach, the application of an input signal on BLs interacts with the SRAM cells column-wise and is used for digital signal domain IMC. Figure 5c is widely used for customized mixed and digital signal domain IMC. A mixed-signal domain activates multiple rows simultaneously, consequently increasing energy efficiency. Row and column circuitry includes signal domain conversions, i.e., digital to analog and vice versa. However, signal domain conversions pose a bottleneck to the performance of mixed-signal domain SRAM-CIM. Recently, the all-digital signal domain CIM-SRAM has emerged as a solution to the challenges faced by the mixed-signal domain, such as the elimination of signal converters, signal precision, and level quantization. Now, computational circuitry consists of arithmetic units for accumulation and activation for DNN execution.

### 3.2. SRAM CIM Signal Domains

Considering the SRAM-based CIM, memory computations (IMC or NMC) can either be in the mixed analog/digital or all-digital signal domain.

#### 3.2.1. Mixed Analog/Digital SRAM Based CIM

**Figure 6:**
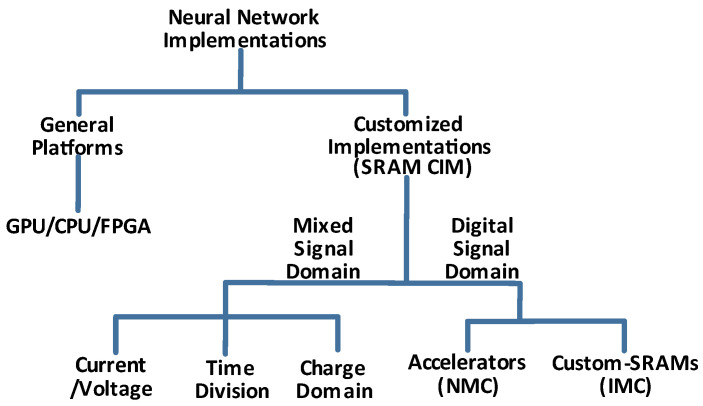
CIM solutions classification according to signal domains.

The mixed-signal domain is energy efficient and provides simultaneous activation of multiple SRAM rows. We can further divide it into three distinct classes, as shown in Figure 6.

- Current/Voltage Domain: An SRAM array stores the initial weight values of neural networks. The input values are carried by either the wordlines (WLs) or bitlines (BLs). In the first case, the WL carries the input signal in the form of amplitude modulation, pulse-width modulation, or pulse frequency [55]. In return, the current flows to/from the multiple SRAM cells into the BLs according to the signal strength present on the WLs. Analog-to-digital converters (ADCs) in peripheral circuits convert the resulting signal voltage on the BLs to the final result.
  In the second case [56], BLs are set to the input values. Afterward, multiple SRAM row activation exposes the corresponding SRAM cells to the BLs. Eventually, a voltage level on the BLs settles to a stable voltage level depending on the weight values stored inside the SRAM cells. Peripheral ADCs convert the settled voltage level to the final digital output value.
- Charge Domain: As opposed to using the BLs or WLs for the input values, the WLs carry ‘1’ or ‘0’ at their nominal levels, but capacitors present at the periphery of SRAM store the resulting charge values. Multiple switching mechanisms [57] control charges into various banks. Signal converters interpret the final value.
- Time Multiplexed Domain: Time domain multiplexers manipulate delay in the final signals’ values with reference to the input and weight values to get the final result. Variable capacitance, current starving, and taping ball tune the delay to perform the compute in memory using time multiplexing [58].

#### 3.2.2. Digital Signal Domain SRAM-Based CIM

As technology scales down, the supply voltage is reduced, which has a major impact on dynamic power consumption. As per the international roadmap for devices and systems (IRDS), the supply voltage would scale down to 600 mV for 0.7 nm node by 2034. At such a low operating voltage, the SRAM-based mixed-signal CIM memory would be challenging for a high-precision NN. In addition, the reduction in voltage headroom (V_DD_-V_th_) will exacerbate the problem of FinFET 6T-SRAM’s reliable operation. Thus, a digital signal domain SRAM is the most suitable candidate for CIM applications.

SRAM being the CIM ultimate choice, we overviewed the recent silicon-verified implementations of convolution neural networks (CNNs). Utilization of multiple databases, i.e., MNIST, AlexNet, VGG, CIFAR, Google-Net, Standford bg, and GTRSB, depicts NN inference with the help of numerous structures. Input and weight precisions of 8b and 1b-16b are taken into consideration, as they achieve the required NN inference and functions. We mention performance evaluation in Table 2, Table 3 and Table 4.

##### Accelerators (NMC)

Table 2 reports five CIM accelerators: Brien, Eyeriss, deep neural processing unit (DNPU), Envision, and Quest. Performance and energy efficiency are critical parameters for a particular design evaluation. These are measured in tera/gega operations per second (T/GOPS) and tera/gega operations per second per watt (T/GOPS/W), respectively. These accelerators have limited weight precision compared with the ones reported in Table 3. BRein [59] uses ternary (0, 1, −1) and binary (0, 1) precision for the weights and input signals, respectively. Layer-wise input/output parallel computation concept successfully incorporates three-layer NNs for a single hardware unit. On-chip functions XNOR, accumulation, and sign achieve MAC and activation functions. Overall, the hardware has 6 processing units in cascade to host 13-layer NNs, without any external data access. Eyeriss [60] has an on-chip memory composed of SRAM cells and an off-chip DRAM memory. These memories hold weights, image features, and their partial sums. The processing element array controls the data flow by adapting to the multiple CNN sizes. In addition, the data-gating technique detects image zero data values to skip some computation functions to enhance power efficiency. Dynamic neural processing units (DNPUs) [61] contain four convolutions clusters and one aggregation core inside a convolution processor. Inside each convolution cluster, weight and image memory provide data to the processing elements to perform computations. The aggregation core accumulates partial sums obtained from processing elements and also executes activation functions and other operations. Envision [62] exploits multivoltage and body biasing techniques together with dynamic voltage and frequency scaling. Multiple SRAM data banks provide simultaneous read and write operations at the same instant. These weights and image storage banks provide data to the MAC array for precision-controlled CNN computations. Quest [63] uses 3D-stacked SRAMs, and each stack is accessible in parallel. The high bandwidth of the data transfer bus and independent memory stack access increase the data retrieval capacity. The overall chip contains 24 cores, where 1 core has a sequencer unit to control operation and address generation, a memory controller, a serializer, de-serializer, and a PE array. PE arrays store images and filter weights in SRAM buffers and carry out MAC operations. Activation operation follows MAC operation. Log-quantization reduces the number of MAC operations, consequently saving energy.

Table 3 summarizes features of five silicon-verified SRAM-based CIM implementations with a weight scalability of 1–16 bits, i.e., high weight precision. A unified neural processing unit (UNPU) [64] has the capability to accelerate CNNs and recurrent NNs with a weight scalability of 1–16 bits. Precision tradeoffs occur among accuracy and energy optimization. The hardware consists of four DNN cores, an aggregation core, and a controller. Each DNN core possesses image features and weights in memory banks and passes on weights to the look-up table processing element for MAC operation. All DNN cores transfer intermediate results to the aggregation core for postprocessing and finalization. The recognition processor [65] core component is the neuron processing engine (NPE) to compute dual-range MAC operations, activation function, and other essential calculations. Furthermore, data compression and external memory access reduction via on-chip memory enhance energy optimization. The origami accelerator [66] exploits scalability for power efficiency. Image and filter banks store features and CNN weights, respectively. Parallel sum-of-products (SoP) units get data from SRAM-based storage banks. An SoP unit is composed of multipliers and adder units to carry out MAC operations, and then it pushes the data onto channel sum units after processing for accumulation completion. Reconfigurable and hybrid NN processors [67] support runtime reconfiguration and partition to adapt themselves as per convolution layer demand. Two 16 × 16 processing element (PE) arrays, memory blocks for images and weights, and controller for data flow are the core components of the processor. Each PE performs all key CNN computations locally and stores the result back in the memory. Precision scalable processor [68] hardware has 256 parallel processing MAC units capable of precision and voltage scaling for low-power operation. An MAC unit passes partial sum results to the on-chip computation unit, which applies more mathematical functions to complete the NN inference. Beforehand, the data compression unit provides convolution sparsity to reduce the number of data bits.

##### Custom SRAM-Based CIM (IMC)

The aforementioned (Table 2 and Table 3) CIMs are ASIC-based accelerators, with the 6T-SRAM memory only being used as storage for weights or input features. However, Table 4 provides custom CIM solutions, where a modified SRAM cell performs some computations right inside the SRAM cell or on the BLs. Computation circuitry at the periphery carries out the remaining arithmetic operations. Colonnade [69] architecture proposes a CIM SRAM cell equipped with a conventional 6T-SRAM cell, a customized XNOR gate, two 2:1 MUXs, and a full adder. This cell, with full adder activated only mode, scales input precision up to 16 bits. The input application at BLs calculates MAC value column-wise, and each result is then passed on to other columns. At the periphery, the post accumulator unit takes partial sum results and produces complete MAC calculations. Compute-SRAM [70] employs 8T transposable SRAM cells, with separate compute WLs and BLs. Conventional read/write operation and associated peripheral circuits remain intact. Moreover, this CIM-SRAM can perform basic Boolean functions and floating-point arithmetic. All-digital CIM-SRAM [71] associates a two-input NOR gate with each SRAM cell. The NOR gate takes one input from the internal node of a 6T-SRAM cell (weight-bit), whereas the input driver provides the second input. This way, the NOR gate multiplies two bits. An adder tree, based on alternate 24T and 28T full adders, takes bit multiplication results and renders MAC operation completion. Multifunctional CIM SRAM [72] employs 7T-SRAM cells, as this cell provides isolation from the internal storage nodes. Alongside this, each SRAM cell carries six additional transistors. Every SRAM column has a dedicated ripple carry adder and multiplier unit for the multiplication and addition operation. A write-back mechanism stores results back into the SRAM. In [73], the SRAM synaptic array stores images and weights in the SRAM array. Adders and registers aid in the row-by-row summation operation. Reduction in weight precision up to ternary level along supply voltage reduction provides low-power operation.

## 4. FinFET 6T-SRAM Cell

This section provides 6T-SRAM cell evolution, i.e., the same as the transistor structural evolution, and some key performance parameters. Afterward, we provide a comprehensive analysis of FinFET 6T-SRAM cell reliability issues.

### 4.1. FinFET 6T-SRAM Cell Design

Conventional 6T-SRAM cells (Figure 4) are still an appealing choice for cache memory mass production due to the minimum number of transistors, dual port for read and write operations, and less leakage current as compared with 7T and 8T SRAM cells. Alternate cells have an edge in noise margins, internal node isolation, and half-cell selection issues but at the expense of increased peripheral circuits, operational complexity, extra control signals, and cell area [74]. However, the FinFET 6T-SRAM differs in transistor structure from the planar CMOS. Therefore, it poses specific challenges to SRAM cell performance.

**Figure 7:**
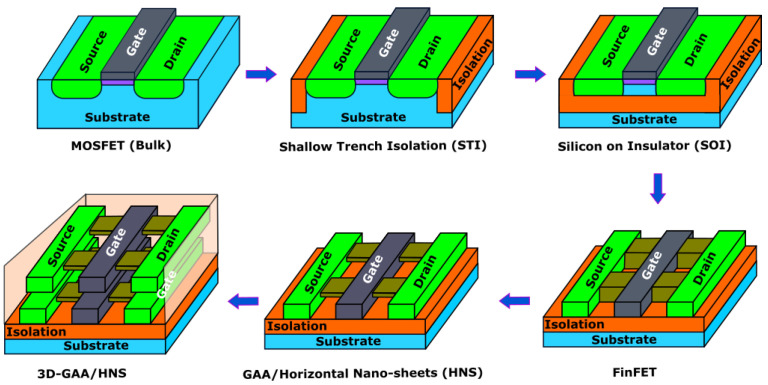
Transistors’ structural evolution from planar CMOS to 3D-stacked GAA transistors.

Figure 7 overviews the evolution of the SRAM cell from a transistor structural perspective. In 1971, Intel introduced the 4004 microprocessors based on 12 µm channel length transistors [75]. Later, in the early 1980s, polycide-gate and silicide resolved issues of the increasing gate and source/drain resistance [76]. In the following decade, in the 1990s, shallow trench isolation (STI) improved the electrical isolation of a device [77]. Then, high-K metal gate (HKMG) and dielectric reduced gate oxide thickness and leakage current [78]. Silicon-on-insulator (SOI) was a major structural modification to mitigate body effect and junction capacitances [79]. A better subthreshold slope owing to better control over the conduction channel led to the modern FinFET design [80]. However, gate all around (GAA) or horizontal nanosheet (HNS) transistors seem to have replaced FinFET due to all-around gate control capability, but they are still being researched. Transistors have scaled down from 12 µm to 3 nm over the last five decades [81]. The 6T-SRAM cell has evolved in parallel to transistor evolution by adopting evolved structural modifications in the transistor.

Table 5 reports performance parameters [82,83,84]. We investigated the FinFET 6T-SRAM cell performance and associated challenges, taking these parameters as the benchmark. Moreover, we used a 256 × 128 FinFET 6T-SRAM array configuration in 12 nm FinFET process node in Cadence Virtuoso for simulations and performance evaluation purposes. In Section 4.2, figures and tables highlight the performance evaluation as per the criteria reported in Table 1.

### 4.2. FinFET 6T-SRAM Cell Reliability

#### 4.2.1. Transistor Sizing

**Figure 8:**
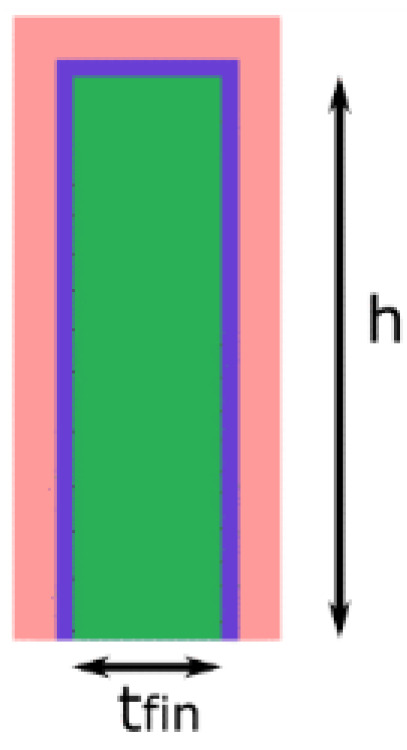
Cross-sectional view of FinFET finger.

Figure 8 shows the Fin cross-sectional view inside a FinFET structure. Two fundamental parameters affecting the FinFET 6T-SRAM performance are the number of fingers used in the gate and source/drain. The FinFET technological node has no stringent control over transistor width. The only choice is a discrete finger number. Equation (1) shows the number of fingers in relation to the transistor width, where h is the fin height, t is the fin thickness, and n shows the number of fins.

(1) $$W=n(2h+t_{fin})$$

Hence, FinFET 6T-SRAM cells are no longer general purpose; they are either area-, performance-, or power-centric. In the literature [85], three different FinFET 6T-SRAM cells exist: high-density (HD), high-performance (HP), and high-current (HC). The transistor sizing ratio for each of them is PU:AC:PD of 1:1:1, 1:1:2, and 1:2:2 for HD, HP, and HC, respectively. Here, PU, AC, and PD represent pull-up, access, and pull-down transistors, respectively. The HD cell uses minimum-size transistors, the HP cell offers better readability with an improved cell ratio, and the HC cell has increased writability due to increased pull ratio.

Furthermore, transistor sizing quantization affects other performance parameters, as listed in Table 6. Here, the HD cell is efficient for static and dynamic power consumption owing to the lower transistor sizes, which limit current flow. The PU and AC sizes in the HP cell make it suitable for the read operation, yet the leakage power becomes double that of the HD cell. The PU and AC strengths in the HC cell keep write time the lowest but degrade the read performance of the HC cell.

**Figure 9:**
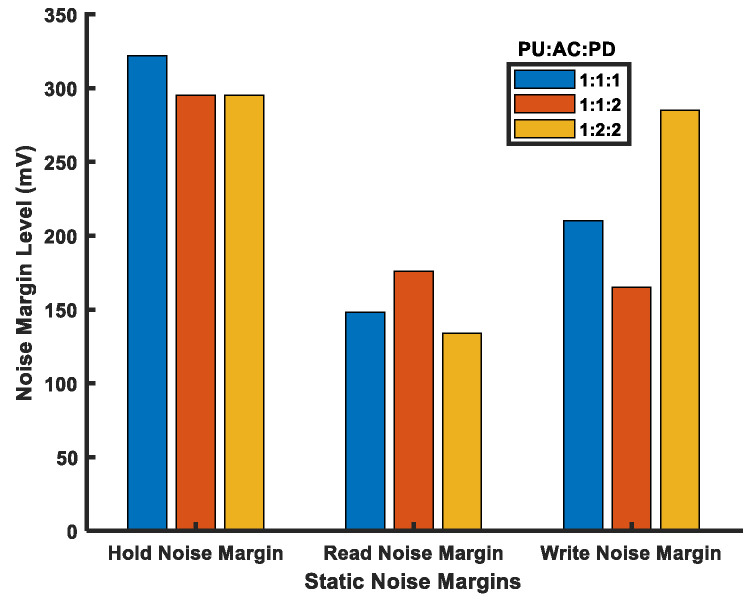
Static noise margins of FinFET 6T-SRAM cells (at V_DD_ = 800 mV).

Figure 9 shows noise margins for the FinFET 6T-SRAM cells. The hold static noise margin (HSNM) is same in the HP and HC configurations because of the same back-to-back inverter strengths, i.e., the PU and PD transistors. The inverter pair in the HD cell has equal PU and PD transistor sizes that keep each inverter switching voltage near the midpoint of supply voltage (V_DD_/2). Thus, its HSNM is higher compared with the HP and HC configurations.

In the read static noise margin (RSNM), the HP cell outperforms due to the stronger PD device compared with the AC device, while the HC cell RSNM suffers from strong AC transistors, as BLs try to impose external value, but the PD transistors oppose it. Whereas the HD cell shows lower RSNM in contrast with the HC cell due to the same strength of the AC and PD transistors, the HC cell is superior in the write static noise margin (WSNM) due to its better pull ratio. However, the cell ratio in the HP and HD cells decreases their WSNMs.

#### 4.2.2. Supply Voltage

**Figure 10:**
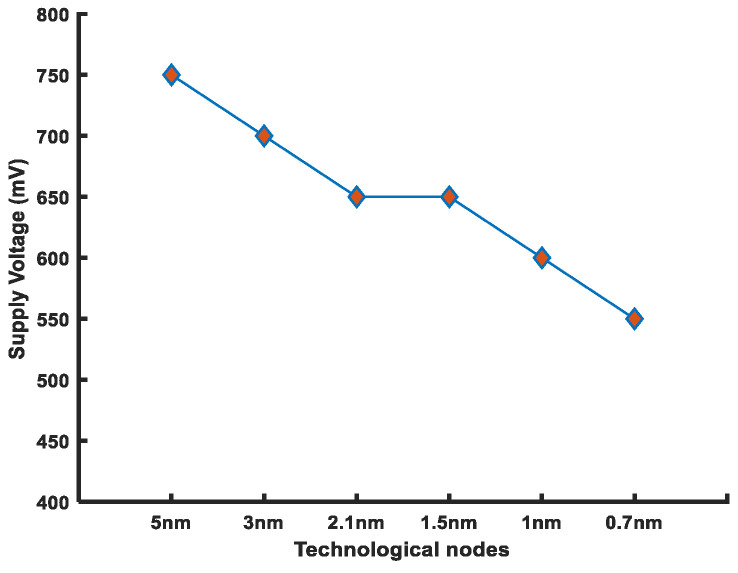
Reduction in supply voltage as a result of technology scaling (IRDS 2022 guidelines).

Noise margins and read/write performance have a direct relation to the supply voltage. Hence, reduced supply voltage poses a serious threat to SRAM-cell stability. The FinFET 6T-SRAM cell has reduced supply voltage; therefore, it is more prone to failures. Figure 10 shows the decreased headroom available for the noise margins. The threshold voltage for the same technological nodes lies between 211–237 mV [81]. Thus, the noise margin will decrease for future technologies. However, the supply voltage reduction improves the power consumption drastically, since the dynamic power depends on the square of the supply voltage.

Table 7 shows the performance variations with respect to the supply voltage. We considered the HD FinFET 6T-SRAM cell to investigate its performance. The value of RSNM is 26 mV (same as to the thermal voltage value) at 600 mV supply voltage. Supply voltage variations beyond this point make cell read operations unstable, hence 600 mV is the minimum operational voltage. As the supply voltage varies between 800–600 mV, the leakage power improves by almost 40% at the cost of increased read (95%) and write (38%) access times. The noise margin determines the minimum operational voltage, whereas power and performance tradeoffs are based on the supply voltage variations.

#### 4.2.3. Threshold Voltage (*V_th_*)

Threshold voltage (*V_th_*) variation has increased in the FinFET 6T-SRAM cell. Variation in *V_th_* is due to the pronounced short-channel effects (*SCE*) and drain-induced barrier lowering (DIBL). Equation (2) shows the *SCE* and *DIBL* relationship with the *V_th_*, where the *V_th_*_∞_ is the nominal value. Applied voltages and electric fields contribute to the *SCE* and *DIBL* [86], thus affecting the *V_th_* nominal value.

(2) $$V_{th}=V_{th\infty }-SCE-DIBL$$

Equation (3) [87] explains *V_th_* variations through the FinFET geometrical parameters, where A is the material-dependent parameter, while $t_{ox}$ and $\varepsilon_{ox}$ represent oxide thickness and permittivity, respectively. *W* and *L* denote the FinFET width and gate length, respectively. As a consequence of the FinFET shrinking dimensions, variation in *V_th_* is not negligible anymore.

(3) $$\sigma_{V_{th}}=A\frac{t_{ox}}{\varepsilon_{ox}}\frac{1}{\sqrt{3WL}}$$

Moreover, any single fin in the FinFET structure (Figure 8) shows different *V_th_* voltages at the center and corner. The difference in the gate work function at these locations is held accountable for this phenomenon [87].

**Figure 11:**
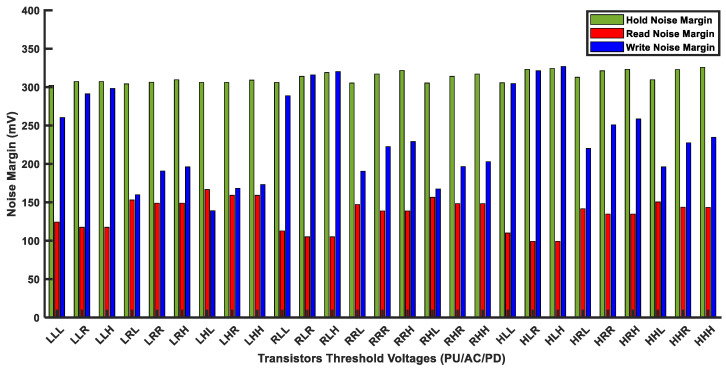
Noise margin measurement of multithreshold FinFET 6T-SRAM HD cells.

In order to analyze *V_th_* effects, we implemented the multithreshold voltage-based (multi-Vth) HD FinFET 6T-SRAM cell. As the FinFET 6T-SRAM cell has 6 transistors, 27 multithreshold SRAM cell combinations are possible by using low-, standard-, and high-threshold voltage FinFET models. As each FinFET model name suggests, the *V_th_* voltage is either low, standard, or high in comparison with one another. Figure 11 shows noise margin measurement under threshold voltage variations. Three alphabets (on the x-axis) for each combination are in order of pull-up, access, and pull-down transistor threshold voltage strengths, respectively. The H, R, and L in Figure 11 denote high-Vth, regular-Vth, and low-Vth voltages, respectively. For example, the RLH combination represents pull-up transistors with regular *V_th_* (R), access transistors with low *V_th_* (L), and pull-down transistors with high *V_th_* (H) strength. We simulated the FinFET 6T-SRAM cell array with a size of 32KB designed with 12 nm FinFET process technology. The noise margin measurements are taken using the butterfly curve method [6].

Out of 27 multi-*V_th_* designs, the RSNM has the lowest noise margin value, while the HSNM has the highest one. Therefore, the RSNM is more sensitive to *V_th_* variations. For HLR and HLH combinations, the RSNM value is just about 100 mV, thus limiting voltage scaling for low-power applications. However, the HSNM value remains above 300 mV for all designs, and the WSNM varies from160 mV to 320 mV. Although the FinFET 6T-SRAM cell has sufficient noise margin for hold, read, and write operations, this can be challenging in conjunction with other issues.

**Figure 12:**
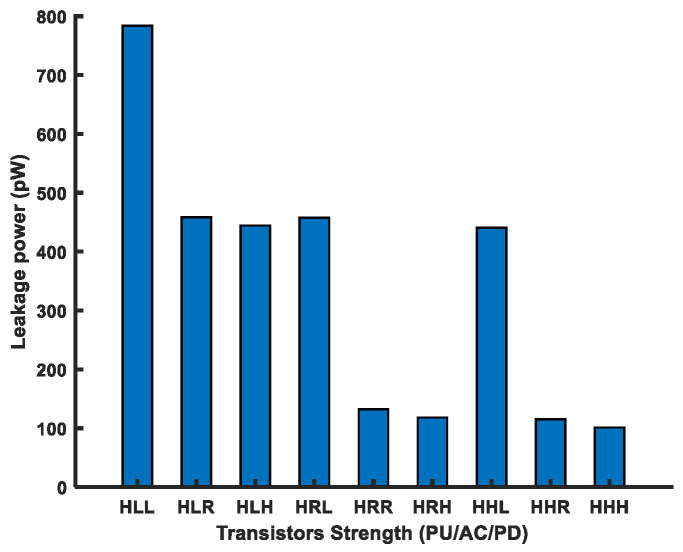
Leakage power of multithreshold FinFET 6T-SRAM HD cells.

As the PU transistor should be the weakest among the other two transistors in a FinFET 6T-SRAM cell, we used the PU transistor with the highest (H) *V_th_* and varied the remaining two transistors’ strengths, i.e., the AC and PD, to analyze cell performance. Figure 12 shows the leakage power for the above-mentioned transistor combinations in the FinFET 6T-SRAM cell. Overall, the leakage power consumption has an inverse relationship with the threshold voltage.

**Figure 13:**
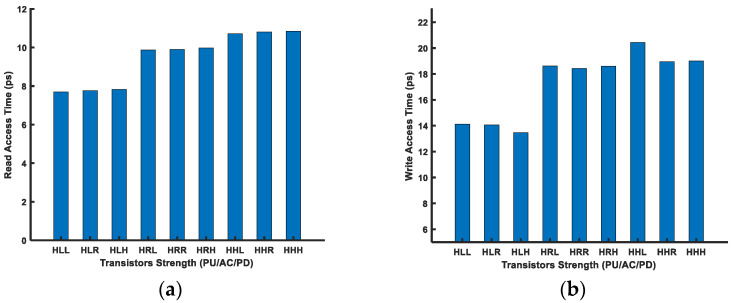
(**a**) Read and (**b**) write performance of a FinFET-SRAM cell for multi-*V_th_* voltages.

Figure 13 shows the read and write access time for the FinFET 6T-SRAM cells. The read time is lower for the cases where the transistor strength difference between the access and pull-down transistor is greater. On the other hand, the write performance is better for the stronger access transistor compared with the pull-up transistor strength. Similarly, the dynamic power consumption will be greater for strong transistors, because they provide more current. For example, dynamic power for HLL would be higher than HRR due to high current driving capabilities.

#### 4.2.4. Temperature and Process Variations

**Figure 14:**
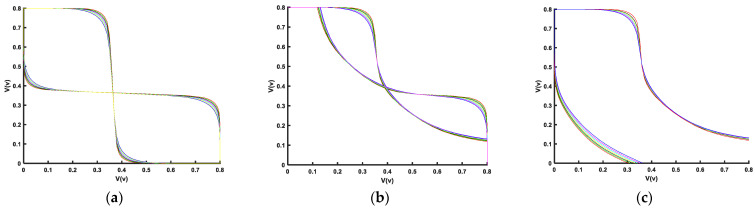
FinFET 6T-SRAM cell noise margins under temperature variation (−40 °C to 125 °C): (**a**) hold noise margin, (**b**) read noise margin, and (**c**) write noise margin.

Due to the extreme geometrical dimensions and material properties of the FinFET, SRAM cell reliability has become a major concern. Previous subsections show the effects of the supply voltage, threshold voltage (*V_th_*), and sizing on the FinFET 6T-SRAM cell performance. This section explores reliability under temperature and process variations. Figure 14 illustrates the effect of temperature on noise margins.

The temperature range is from −40 °C to 125 °C. HSNM (Figure 14a), RSNM (Figure 14b), and WSNM (Figure 14c) show variations of about 13%, 22%, and 12%, respectively. This variation is with reference to the noise measurement at room temperature i.e., 27 °C. Here, RSNM is more important, as it is the lowest of the two noise margins. Nonetheless, it remains above 115mV. The variation in noise margin is due to the current carrying variations because of temperature difference.

**Figure 15:**
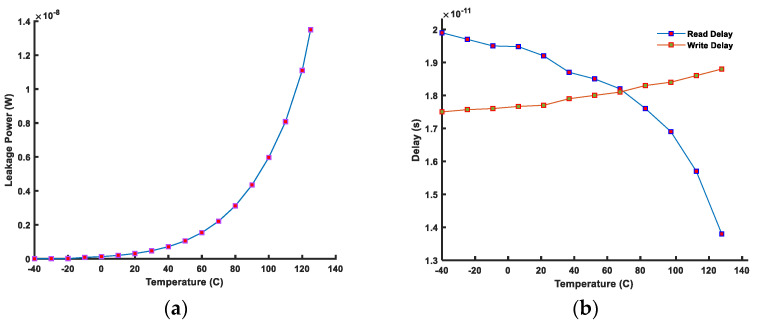
(**a**) Leakage power and (**b**) read/write performance of a FinFET 6T-SRAM cell under temperature variations.

Figure 15 shows power and performance evaluation. The leakage power remains at about the same level till room temperature, i.e., 27 °C, as shown in Figure 15a. However, it increases exponentially after 60 °C due to a sudden increase in leakage current. The effect of temperature on read and write delays is shown in Figure 15b. The read delay decreases as the temperature increases. This is because an increase in temperature decreases the transistor current due to the carrier mobility degradation. A decrease in the transistor current at high temperature causes BLs capacitances to charge to a lower level, thus decreasing the discharge time. Consequently, this reduces the read delay and read power consumption at high temperatures, whereas the write performance is opposite to the read performance. An increase in the temperature decreases the transistor current; thus, accordingly, the internal cell nodes take more time to change their stored logic level.

A 6T-SRAM cell’s performance varies with the FinFET process deviations. Table 8 reports performance parameters under NFET and PFET variations. The read performance is worse for the slower NFET because of performance degradation in the pull-down transistor. Similarly, the write performance shows improvement for the fast PFET due to the increased access transistor strength. Improvement in the cell and pull-up ratio improves the read and write performances, respectively, and vice versa. On account of the maximum transistor strength difference, the RSNM and WSNM show their worst tolerance for FS and SF process corners, respectively.

## 5. Conclusions

Modern AI requires deep neural networks (DNNs), whose big data requirement is bottleneck by SRAM performance. CIM solves this challenge by reducing off-chip access. We reviewed silicon-verified all-digital domain SRAM-based accelerators and custom SRAM-based CIM solutions, focusing on multiply and accumulate (MAC) operations for multiple performance measurement benchmarks. ASIC-based CIM solutions utilize 8b input precession, whereas weight precision ranges from 4b–16b. The maximum operational frequency reported is 500 MHz. The highest energy efficiency achieved is 156 TOPS/W, with an area efficiency of 6750 GOPS/mm^2^.

We investigated the basic building block’s performance, i.e., the FinFET 6T-SRAM cell, taking noise margins, read operation, and write operation as performance measurement benchmarks. Variations in FinFET sizing, threshold voltage, supply voltage, process, and environmental conditions make the FinFET 6T-SRAM cell unable to achieve the simultaneous optimization of area, power, and performance. Therefore, a cell is either high-density (HD), high-performance (HP), or high-current (HC) to achieve design-specific targets. A comprehensive investigation puts forth FinFET 6T-SRAM cell evaluations and limitations under various processes and operational and environmental conditions. Improvement in one parameter degrades other performance parameters. HC cell configuration shows a write access time of 9.17 ps (1.31 times more efficient than the HD configuration), whereas read and write power consumptions are the lowest for the HD configuration. The HSNM margin is above 300 mV for all cell configurations. Under supply voltage variations, RSNM goes down to 26 mV at 600 mV, which is a major concern. Under temperature variations, write delay increases, but read delay decreases by up to 25% as a result of decreased current driving capability.

## Figures and Tables

**Table 1:** Memory cell performance evaluation as guideline for potential CIM application choice [11,12,13,14,15,16,17,18,19,20,21,22,23,24,25,26,27,28,29,30,31,32,33,34,35,36,37,38,39,40,41,42,43,44,45,46].

Parameter	6T-SRAM	DRAM	ReRAM	NAND Flash	NOR Flash	PCM	FRAM	MRAM	FeFET
Transistor Num.	6	1	1	1	1	1	1	1	1
Supply Voltage	<1 V	~1 V	~3 V	<10 V	<10 V	<3 V	<4 V	<2 V	<4 V
Read Time	1 ns	10 ns	<10 ns	50 ns	10 ns	<10 ns	<150 ns	<10 ns	<8 ns
Write Time	1 ns	10 ns	<10 ns	0.01–1 ms	0.1–10 ms	50 ns	<50 ns	<10 ns	<10 ns
Leakage Power	<35 pW	<12 pW	<5 pW	<1 pW	<2 pW	<10 pW	<4 pW	<4 pW	<5 pW
Write Operating Voltage	<1 V	<1 V	<3.2	<10	<9	<3 V	<4	<2	<4
Read Operating Voltage	<1 V	<1 V	<1.5	<9	<10	<3 V	<3.3 V	<1.2	<3.5
Retention	N/A	64 ms	>10 y	>10 y	>10 y	>10 y	>10 y	>10 y	>10 y
Endurance	>10^16^	>10^16^	10^8^–10^12^	>10^4^	>10^5^	10^8^–10^15^	>10^10^	>10^15^	>10^10^
Volatility	Yes	Yes	No	No	No	No	No	No	No
Maturity	3 nm (FinFET)	10 nm	22 nm	20 nm	22 nm	20 nm	28 nm	22 nm	22 nm

**Table 2:** SRAM-based digital signal domain CIM accelerators with low weight precision [59,60,61,62,63].

	BRein [59]	Eyeriss [60]	DNPU [61]	Envision [62]	Quest [63]
Technology	65 nm	65 nm	65 nm	28 nm (FD-SOI)	40 nm
Supply Voltage(V)	1	1.17	1.1	1	1.1
SRAM Capacity	12 × (69 KB)	181.5 KB	290 KB	128 KB	7680 KB
CIM Function	MAC and Activation	MAC and Activation	MAC and Activation	MAC and Activation	MAC and Activation
Input Precision	1b	8b	8b	8b	8b
Algorithms	MLP/CNN	CNN	CNN/RNN	CNN	CNN/RNN/MLP
Database	MNIST	AlexNet	ImageNet(VGG)	AlexNet/VGG	AlexNet/VGG
Weight Precision	+1/0/−1	1–12b	4/8/16b	1–8b	1–4b
Type	Accelerator (PIM)	Accelerator (PE)	Processor	Processor	Inference Engine
Clock Frequency	400 MHz	250 MHz	200 MHz	200 MHz	330 MHz
Performance	1.3 TOPS	42 GOPS	1.2 TOPS	300 GOPS	7.49 TOPS
Energy Efficiency	6 TOPS/W	-	8.1 TOPS/W	10 TOPS/W	-
Area Efficiency	0.365 (TOPS/mm^2^)	-	-	-	-
Silicon Verified	Yes	Yes	Yes	Yes	Yes
	1MAC = 2 OPS	1 MAC = 1 OPS	-	-	-

**Table 3:** SRAM-based digital signal domain CIM accelerators with higher weight precision [64,65,66,67,68].

	UNPU [64]	Recognition [65]	Origami [66]	Hybrid [67]	Scalable [68]
Technology	65 nm	65 nm	65 nm	65 nm	40 nm
Supply Voltage(V)	1.1	1.2	1.2	1.2	1.1
SRAM Capacity	256 KB	36 KB	344 KB	348 KB	148 KB
CIM Function	MAC and Activation	MAC and Activation	MAC and Activation	MAC and Activation	MAC and Activation
Input Precision	8b	8b	8b	8b	8b
Algorithms	CNN	CNN	CNN	CNN/RCNN/FCN	CNN
Database	AlexNet/ GoogleNet	MNIST/CIFAR/ AlexNet/GTSRB	MNIST/Cifar/ Standford Bg	AlexNet/LRCN	AlexNet/LeNet
Weight Precision	1–16b	1–16b	1–16b	1–16b	1–16b
Type	Accelerator	Processing Engine	Accelerator	Accelerator	Processor
Clock Frequency	200 MHz	125 MHz	500 MHz	200 MHz	204 MHz
Performance	7372 GOPS	64 GOPS	196 GOPS	409.6 GOPS	102 GOPS
Energy Efficiency	50.6 TOPS/W	1.42 TOPS/W	803 GOPS/W	5.09 TOPS/W	1.75 TOPS/W
Area Efficiency	-	-	23.9 GOPS/mm^2^	-	-
Silicon Verified	Yes	Yes	Yes	Yes	Yes
	-	1 MAC = 2 OPs	-	-	-

**Table 4:** Custom SRAM-cell-based digital signal domain CIM for deep neural network accelerations [69,70,71,72,73].

	Colonade [69]	Compute [70]	A-Digital [71]	Multifunc. [72]	Synaptic [73]
Technology	65 nm	28 nm	22 nm	40 nm	65 nm
Supply Voltage(V)	0.8	1.1	0.8	0.9	1
SRAM Capacity	16 KB	16 KB	64 KB	1 KB	2 KB
CIM Function	MAC	MAC and Boolean	MAC	MAC and Boolean	MAC
Input Precision	1–16b	1–8b	1–8b	1b/4b	1b
Algorithms	CNN	CONV/FC/FIR/ GRAPH	CNN	TNN	CNN
Database	LeNET/Alex- Net/VGG	Cuda/AlexNet/Tap Filter/Neighbor	-	-	MNIST
Weight Precision	1–16b	8b	4/8/12/16b	+1/0/−1	+1/0/−1
Type	Custom- SRAM	Custom- SRAM	Custom- SRAM	Custom- SRAM	Custom- SRAM
Clock Frequency	135 MHz	475 MHz	100 MHz	100 MHz	1 GHz
Performance	567 GOPS	32.7 GOPS	3300 GOPS	5.6 GOPS	-
Energy Efficiency	156 TOPS/W	5.27 TOPS/W	89 TOPS/W	7.66 TOPS/W	-
Area Efficiency	6750 GOPS/mm^2^	27.3 GOPS/mm^2^	16.3 TOPS/mm^2^	27 GOPS/mm^2^	-
Silicon Verified	Yes	Yes	Yes	Yes	Yes

**Table 5:** Performance parameters for the FinFET 6T-SRAM cell performance evaluation.

Parameter	Measurement
Noise Margins	Margin to ensure SRAM cell operation in hold/read/write modes
Read Time	Wordline low-to-high transition to 200 mV bitlines voltage difference
Write Time	Wordline low-to-high transition to 90% or 10% of stored value
Leakage Power	Leakage power under hold mode (average of holding 0 and 1 value)
Read/Write Power	Power consumption under read and write operational mode

**Table 6:** Performance parameters of 6T FinFET-SRAM cells.

Performance Parameter	Cell Configuration (PU:AC:PD)		
	HD (1:1:1)	HP (1:1:2)	HC (1:2:2)
Read Access Time (ps)	1.11	1.08	1.47
Write Access Time (ps)	21.2	13.2	9.17
Leakage Power (pW)	414	867	869
Read Power (µW)	9.15	9.84	13.7
Write Power (µW)	7.43	23	22.8

**Table 7:** FinFET-SRAM cell performance evaluation under supply voltage variations (temperature = 27 °C).

Supply Voltage (mV)	Performance Parameters							
	Read Access Time (ps)	Write Access Time (ps)	Read Dynamic Power (µW)	Write Dynamic Power (µW)	Leakage Power (pW)	HSNM (mV)	RSNM (mV)	WSNM (mV)
800	1.11	21.2	9.15	7.43	464	323	141	220
750	1.26	23.4	8.61	6.73	413	298	113	198
700	1.43	24.7	7.50	5.91	364	279	82	174
650	1.76	26.5	6.47	5.09	320	254	53	151
600	2.17	29.1	5.51	4.39	281	231	26	129

**Table 8:** FinFET-SRAM cell reliability analysis under corner cases (F = Fast, S = Slow, T = Typical).

Corner Case NFET/PFET	Performance Parameters							
	Read Access Time (ps)	Write Access Time (ps)	Read Dynamic Power (µW)	Write Dynamic Power (µW)	Leakage Power (pW)	HSNM (mV)	RSNM (mV)	WSNM (mV)
TT	11.82	18.12	10.4	1.89	21.8	323	141	220
SS	14.89	21.92	20.3	1.49	9.81	337	150	224
SF	14.26	23.5	10.15	2.14	15.9	309	172	137
FS	9.46	15.61	10.6	1.76	10.08	307	97	286
FF	9.6	16.06	11.03	2.51	62.8	293	119	216

## Data Availability

Data can be provided on request.

## References

[1] Sze V., Chen Y., Yang T., Emer J. (2017). Efficient Processing of Deep Neural Networks: A Tutorial and Survey. Proc. IEEE.

[2] (2021). AI Acceleration: Autonomous is driving by Manouchehr Rafie VP of Advance Technologies.

[3] Le Y., Wang Z., Liu Y., Chen P. (2021). The Challenges and Emerging Technologies for Low Power Artificial Intelligence IoT Systems. IEEE Trans. Circuit Syst. -I Regul. Pap..

[4] Verma N., Jia H., Valavi H., Tang Y., Ozatay M., Chen L., Zhang B., Deaville P. (2019). In Memory computing: Advances and prospects. IEEE Solid State Circuit Mag..

[5] Yu S., Jiang H., Huang S., Peng X., Lu A. (2021). Compute-in-Memory Chips for Deep learning: Recent Trends and Prospects. IEEE Circuit Syst. Mag..

[6] Qazi M., Sinangil M., Chandrakasan A. (2010). Challenges and Directions for Low Voltage SRAM. IEEE Des. Test Comput..

[7] Turi M.A., Delgado-Frias J.G. (2020). Effective Low Leakage 6T and 8T FinFET SRAMs: Using Cells with Reverse Biased FinFETs, Near Threshold Operation and Power Gating. IEEE Trans. Circuits Syst. II Express Brief.

[8] Parasad G., Mand B.C., Ali M. (2021). Soft Error Aware SRAM for Terrestrial Applications. IEEE Trans. Device Mater. Reliab..

[9] Saeidi R., Nabavi M., Savaria Y. SRAM security and vulnerability to Hardware Trojan: Design Considerations. Proceedings of the IEEE 63rd International Conference of Midwest Symposium on Circuits and Systems (MWSCAS).

[10] International Roadmap for Device and Systems (IRDS) 2022. https://irds.ieee.org/editions/2022.

[11] Song T., Kim H., Rim W., Jung H., Park C., Lee I., Baek S., Jung J. (2022). A 3-nm Gate-All-Around SRAM Featuring an Adaptive Dual-Bitline and an Adaptive Cell-Power Assist Circuit. IEEE J. Solid State Circuits.

[12] Chang T.Y.J., Chen Y.H., Chan G., Cheng H., Wang P.S., Lin Y., Fujiwara H., Lee R., Liao H.J., Wang P.W. (2021). A 5-nm 135-Mb SRAM in EUV and High-Mobility Channel FinFET Technology with Metal Coupling and Charge-Sharing Write-Assist Circuitry Schemes for High-Density and Low-VMIN Applications. IEEE J. Solid State Circuits.

[13] Cho K., Park J., Kim K., Oh T.W., Jung S.O. (2021). SRAM Write Assist Circuit Using Cell Supply Voltage Self Collapse with bitline Charge Sharing for Near Threshold Operation. IEEE Trans. Circuit Syst. II.

[14] Wang J., An H., Zhang Q., Kim H.S., Blaauw D., Sylvester D. (2020). A 40-nm Ultra-Low Leakage Voltage-Stacked SRAM for Intelligent IoT Sensors. IEEE Solid State Circuits Lett..

[15] Chun K.C., Kim Y.K., Ryu Y., Park J., Oh C.S., Byun Y.Y., Kim S.Y., Shin D.H., Lee J.G., Ho B.K. (2021). A 16-GB 640-GB/s HBM2E DRAM with a Data-Bus Window Extension Technique and a Synergetic On-Die ECC Scheme. IEEE J. Solid State Circuits.

[16] Kim Y.H., Kim H.J., Choi J., Ahn M.S., Lee D., Cho S.H., Park D.Y., Park Y.J., Jang M.S., Kim Y.J. A 16Gb Sub-1V 7.14Gb/s/pin LPDDR5 SDRAM Applying a Mosaic Architecture with a Short-Feedback 1-Tap DFE, an FSS Bus with Low-Level Swing and an Adaptively Controlled Body Biasing in a 3rd-Generation 10nm DRAM. Proceedings of the IEEE International Conference on Solid State Circuits (ISSCC).

[17] Son M., Jung S.G., Kim S.H., Park E., Lee S.H., Yu H.Y. (2021). Enhancement of DRAM Performance by Adopting Metal–Interlayer–Semiconductor Source/Drain Contact Structure on DRAM Cell. IEEE Trans. Electron Devices.

[18] Wong H.S.P., Raoux S., Kim S., Liang J. (2010). Phase Memory. Proc. IEEE.

[19] Imran M., Kwon T., Yang J.S. (2022). ADAPT: A Write Disturbance-Aware Programming Technique for Scaled Phase Change Memory. IEEE Trans. Comput. Aided Des. Integr. Circuits Syst..

[20] Chang C.W., Wu C.F., Chang Y.H., Yang M.C., Chang C.F. (2022). Leveraging Write Heterogeneity of Phase Change Memory on Supporting Self-Balancing Binary Tree. IEEE Trans. Comput. Aided Des. Integr. Circuits Syst..

[21] Min D., Park J., Weber O., Wacquant F., Villaret A., Vandenbossche E., Arnaud F., Bernard E., Elghouli S., Boccaccio C. 18nm FDSOI Technology Platform embedding PCM & Innovative Continuous-Active Construct Enhancing Performance for Leading-Edge MCU Applications. Proceedings of the IEEE International Electron Devices Meeting (IEDM).

[22] Chih Y.D., Chou C., Shih Y.C., Lee C.F., Khwa W.S., Wu C.Y., Shen K.H., Chu W.T., Chang M.F., Chuang H. Design Challenges and Solutions of Emerging Nonvolatile Memory for Embedded Applications. Proceedings of the IEEE International Electron Devices Meeting (IEDM).

[23] Zhang Y., Yu Z., Gu L., Wang C., Feng D. (2021). EnTiered-ReRAM: An Enhanced Low Latency and Energy Efficient TLC Crossbar ReRAM Architecture. IEEE Access.

[24] Ntinas V., Rubio A., Sirakoulis G.C., Aguilera E.S., Pedro M., Crespo-Yepes A., Martinez J.M., Rodriguez R., Nafria N.M. (2021). Power-Efficient Noise-Induced Reduction of ReRAM Cell’s Temporal Variability Effects. IEEE Trans. Circuits Syst. -II Express Briefs.

[25] Sun W., Lim H., Shin H. Investigation of power dissipation for ReRAM in crossbar array architecture. Proceedings of the IEEE 14th Annual Non-Volatile Memory Technology Symposium (NVMTS).

[26] Xu C., Niu D., Muralimanohar N., Balasubramonian R., Zhang T., Yu S., Xie Y. Overcoming the Challenges of Crossbar Resistive Memory Architectures. Proceedings of the 21st International Symposium on High Performance Computer Architecture (HPCA).

[27] Na D., Na D., Kavala A., Cho H., Lee J., Yang M., Song E., Kim T., Lee S.K., Jang D.S. (2021). A 1.8-Gb/s/Pin 16-Tb NAND Flash Memory Multi-Chip Package with F-Chip for High-Performance and High-Capacity Storage. IEEE J. Solid State Circuits.

[28] Shim W., Yu S. (2021). System-Technology Codesign of 3-D NAND Flash Based Compute-in-Memory Inference Engine. IEEE J. Explor. Solid State Comput. Devices Circuits.

[29] Kim M., Yun S.W., Park J., Park H.K., Lee J., Kim Y.S., Na D., Choi S., Song Y., Lee J. A 1Tb 3b/Cell 8th-Generation 3D-NAND Flash Memory with 164MB/s Write Throughput and a 2.4Gb/s Interface. Proceedings of the IEEE Solid State Circuit Conference (ISSCC).

[30] Torsi A., Zhao Y., Liu H., Tanzawa T., Goda A., Kalavade P., Parat K. (2021). A Program Disturb Model and Channel Leakage Current Study for Sub-20 nm NAND Flash Cells. IEEE Trans. Electron Devices.

[31] Coignus J., Torrente1 G., Vernhet A., Renard S., Roy D., Reimbold G. Modelling of 1T-NOR Flash Operations for Consumption Optimization and Reliability Investigation. Proceedings of the IEEE International Reliability Physics Symposium (IRPS).

[32] Kumar M.P., Ganta J.R., Kumar K.S., Vani P.K. An efficient Flash Memory Devices. Proceedings of the IEEE International Conference on Intelligent Systems and Green Technology (ICISGT).

[33] Lue H.T., Hsu T.H., Yeh T.H., Chen W.C., Lo C., Huang C.T., Lee G.R., Chiu C.J., Wang K.C., Lu C.Y. A Vertical 2T NOR (V2T) Architecture to Enable Scaling and Low-Power Solutions for NOR Flash Technology. Proceedings of the IEEE Symposium on VLSI Technology.

[34] Toshiba NAND vs. NOSR Flash Memory Technology Overview. http://atuing.umcs.maine.edu/~meadow/courses/cos335/Toshiba%20NAND_vs_NOR_Flash_Memory_Technology_Overviewt.pdf.

[35] Lin Y.D., Lee H.Y., Tang Y.T., Yeh P.C., Yang H.Y., Yeh P.S., Wang C.Y., Su J.W., Li S.H., Sheu S.S. 3D Scalable, Wake-up Free, and Highly Reliable FRAM Technology with Stress-Engineered HfZrOx. Proceedings of the IEEE International Electron Devices Meeting (IEDM).

[36] Lomenzo P.D., Slesazeck S., Hoffmann M., Mikolajick T., Schroeder U., Max B., Mikolajick T. Ferroelectric Hf1-xZrxO2 Memories: Device Reliability and Depolarization Fields. Proceedings of the IEEE 19th Non-Volatile Memory Technology Symposium (NVMTS).

[37] Lin Y.D., Lee1 H.Y., Tang Y.T., Yeh P.C., Yang H.Y., Yeh P.S., Wang C.Y., Su J.W., Li S.H., Sheu S.S. (2020). Promising Engineering Approaches for Improving the Reliability of HfZrOx 2-D and 3-D Ferroelectric Random Access Memories. IEEE Trans. Electron Devices.

[38] Wei J.N., Guo H.X., Zhang F.Q., Guo G., He C.H. Analysis of SEE modes in ferroelectric random access memory using heavy ions. Proceedings of the IEEE 26th International Symposium on Physical and Failure Analysis of Integrated Circuits (IPFA).

[39] Texas Instruments (TI) Technical Document on FRAM. https://www.ti.com/lit/wp/slat151/slat151.pdf?ts=1657556247596&ref_url=https%253A%252F%252Fwww.google.com%252F.

[40] Khanna S., Bartling S.C., Clinton M., Summerfelt S., Rodriguez J.A., McAdams H.P. (2014). An FRAM-Based Nonvolatile Logic MCU SoC Exhibiting 100% Digital State Retention at 0 V Achieving Zero Leakage With 400-ns Wakeup Time for ULP Applications. IEEE J. Solid State Circuits.

[41] Xiang J., Chang W.H., Saraya T., Hiramoto T., Irisawa T., Kobayashi M. (2021). Ultrathin MoS_2_-Channel FeFET Memory with Enhanced Ferroelectricity in HfZrO_2_ and Body-Potential Control. J. Electron Devices Soc..

[42] Peng H.K., Lai T.C., Kao T.H., Wu Y.H. (2022). Improved Reliability and Read Latency Under Radiation Observed in HfZrOx Based p-FeFETs with AlON Interfacial Layer. IEEE Electron Devices Lett..

[43] Tung C.T., Pahwa G., Salahuddin S., Hu C. (2022). A Compact Model of Ferroelectric Field-Effect Transistor. IEEE Electron Devices Lett..

[44] Kim J.Y., Choi M.J., Jang H.W. (2021). Ferroelectric field effect transistors: Progress and perspective. Appl. Phys. Lett. Mater..

[45] George S., Ma K., Aziz A., Li X., Khan A., Salahuddin S., Chang M.F., Datta S., Sampson J., Gupta S. Non-volatile Memory Design Based on Ferroelectric FETS. Proceedings of the 53rd IEEE International Conference on Design Automation (DAC).

[46] Reis D., Ni K., Chakraborty W., Yin X., TrenTzsch M., Dunkel S., Melde T., Muller J., Beyer S., Datta S. (2019). Design and Analysis of an Ultra-Dense, Low-Leakage, and Fast FeFET-Based Random Access Memory Array. IEEE J. Explor. Solid State Comput. Devices Circuits.

[47] Arun A.V., Sreelekhshmi P.S., Jacob J. (2022). Design and analysis of dopingless 1T DRAM using workfunction engineered tunnel field effect transistors. Microelectron. J..

[48] Sharma D.K., Khosla R., Sharma S.K. (2015). Multilevel metal/Pb(Zr0.52Ti0.48)/TiOxNy/Si fornext generation FeRAM technology node. Solid State Electron. J..

[49] Hadamek T., Siegfried S., Wolfgang G., Viktor S. (2023). Modelling thermal effects in STT-MRAM. Solid State Electron. J..

[50] Yoon D.G., Sim J.M., Song Y.H. (2023). Mechanical Stress in Tappered Channel hole of 3D NAND Flash memory. J. Microelectron. Reliab..

[51] Matteo F., Simola R., Pellerin J.P., Coulie K. (2022). 1T-NOR Flash memory after endurance degradation: An advanced TCAD simulation. J. Microelectron. Reliab..

[52] Dowoon L., Dongjoo B., Sungho K., Kim H.D. (2022). Correlation between resistive switching characteristics and density of traps observed in Zr_3_N_2_ resistive switching memory devices with TiN barrier electrode. Int. J. Ceram..

[53] Wang Q., Niu G., Luo R., Fang W., Wang R., Xu Y., Song Z., Ren W., Song S. (2022). PCRAM electronic synapse measurements based on pulse programming engineering. J. Microelectron. Eng..

[54] Jung J., Lee D., Kim S., Kim H.D. (2021). Self-Rectifying Characteristics Observed in O-Doped ZrN Resistive Switching Memory Devices Using Schottky Barrier Type Bottom Electrode. IEEE Access.

[55] Jhang C.J., Xue C.X., Hung J.M., Chang F.C., Chang M.F. (2021). Challenges and Trends of SRAM-Based Computing-in-Memory for AI Edge Devices. IEEE Trans. Circuits Syst. -I.

[56] Deng L., Li G., Han S., Shi L., Xie Y. (2020). Model Compression and Hardware Acceleration for Neural Networks: A Comprehensive Survey. Proc. IEEE.

[57] Lee E., Han T., Seo D., Shin G., Kim J., Kim S., Jeong S., Rhe J., Park J., Ko J.H. (2021). A Charge-Domain Scalable-Weight In-Memory Computing Macro with Dual-SRAM Architecture for Precision-Scalable DNN Accelerators. IEEE Trans. Circuits Syst..

[58] Song J., Wang Y., Guo M., Ji X., Cheng K., Hu Y., Tang X., Wang R., Huang R. (2021). TD-SRAM: Time-Domain-Based In-Memory Computing Macro for Binary Neural Networks. IEEE Trans. Circuits Syst..

[59] Ando K., Kodai U., Kentaro O., Haruyoshi Y., Shimpei S., Hiroki N., Yamazaki T., Masayuki I.S., Tetsuya A., Tadahiro K. (2018). Brien Memory: A single Chip Binary/Ternary Reconfigurable in-Memory Deep Neural Network Accelerator Achieving 1.4 TOPS at 0.6W. IEEE J. Solid State Circuits.

[60] Chen Y.H., Krishna T., Emer J.S., Sze V. Eyeriss: An Energy Efficient Reconfigurable Accelerator for Deep Convolution Neural Network. Proceedings of the IEEE International Solid State Circuits Conference (ISSCC).

[61] Shin D., Lee J., Lee J., Yoo H.J. DNPU: A 8.1 TOPS/W reconfigurable CNN-RNN processor for general purpose deep neural networks. Proceedings of the IEEE International Solid State Circuits Conference (ISSCC).

[62] Moons B., Uytterhoeven R., Dehaene W., Verhelst M. Envision: A 0.26 to 10 TOPS/W subword-parallel dynamic voltage accuracy frequency scalable Convolution Neural Network processor in 28nm FDSOI. Proceedings of the IEEE International Solid State Circuits Conference (ISSCC).

[63] Ueyoshi K., Ando K., Hirose K., Yamazaki T.S., Kadomoto J., Miyata T., Hamada M., Kuroda T., Motomura M. QUEST: A 7.49TOPS Multi-Purpose Log-Quantized DNN Inference Engine Stacked on 96MB 3D SRAM Using Inductive-Coupling Technology in 40nm CMOS. Proceedings of the IEEE International Solid State Circuits Conference (ISSCC).

[64] Lee J., Kim C., Kang S., Shin D., Kim S., Yoo H.J. (2019). UNPU: An Energy Efficient Deep Neural Network Accelerator with Fully Variable Weight Bit Precision. IEEE J. Solid State Circuits.

[65] Sim J., Park J.S., Kim M., Bae D., Choi Y., Kim L.S. A 1.42TOPS/W Deep Convolutional Neural Network Recognition Processor for Intelligent IoE Systems. Proceedings of the IEEE International Solid State Circuits Conference (ISSCC).

[66] Cavigellie L., Benini L. (2017). Origami: A 803-GOp/s/W Convolutional Network Accelerator. IEEE Trans. Circuits Syst. Video Technol..

[67] Peng S.Y., Ouyang P., Tang S., Tu F., Li X., Liu L., Wei S. A 1.06-to-5.09 TOPS/W Reconfigurable Hybrid-Neural-Network Processor for Deep Learning Applications. Proceedings of the IEEE Symposium on VLSI Circuits.

[68] Moon B., Vershelt M. A 0.3-2.6 TOPS/W precision scalable processor for real time large scale ConvNets. Proceedings of the IEEE Symposium on VLSI Circuits (VLSI-Circuits).

[69] Kim H., Yoo T., Kim T.T.H., Kim B. (2021). Colonnade: A Reconfigurable SRAM-Based Digital Bit-Serial Compute-In-Memory Macro for Processing Neural Networks. IEEE J. Solid State Circuits.

[70] Wang J., Wang X., Eckert C., Subramaniyan A., Das R., Blaauw D., Sylvester D. (2020). A 28-nm Compute SRAM with Bit-Serial Logic/Arithmetic Operations for Programmable In-Memory Vector Computing. IEEE J. Solid State Circuits.

[71] Chih Y.D., Lee P.H., Fujiwara H., Shih Y.C., Lee C.F., Naous R., Chen Y.L., Lo C.P., Lu C.H., Mori H. An 89TOPS/W and 16.3TOPS/mm2 All-Digital SRAM-Based Full-Precision Compute-In Memory Macro in 22nm for Machine-Learning Edge Applications. Proceedings of the IEEE International Solid State Circuit Conference (ISSCC).

[72] Wang C.C., Tolentino L.K.S., Huang C.Y., Yeh C.H. (2021). A 40nm CMOS Multifunctional Compute in Memory Using Single Ended disturb free 7T 1 KB SRAM. IEEE Trans. Very Large Scale Integr. VLSI Syst..

[73] Sun X., Liu R., Chen Y.J., Chiu H.Y., Chen W.H., Chang M.F., Yu S. (2017). Low-VDD Operation of SRAM Synaptic Array for Implementing Ternary Neural Network. IEEE Trans. Very Large Scale Integr. VLSI Syst..

[74] Gul W., Shams M., Al-Khalili D. (2022). SRAM Cell Design Challenges in Modern Deep Sub Micron Technologies: An Overview. Int. J. Micromach..

[75] Patt Y. (2001). Requirements, bottleneck, and good fortune: Agents for microprocessor evolution. Proc. IEEE.

[76] Mann R.W., Clevenger L.A., Agnello P.D., White F.R. (1995). Silicides and local interconnections for high performance VLSI applications. IBM J. Res. Dev..

[77] Nandakumar M., Chatterjee A., Sridhar S., Joyner K., Rodder M., Chen I.C. Shallow trench isolation for advanced ULSI CMOS technologies. Proceedings of the International Electron Devices Meeting (IEDM).

[78] Auth C. 45nm high K + metal gate strain enhanced CMOS. Proceedings of the IEEE Custom Integrated Circuits Conference (CICC).

[79] Lemnios Z.J., Daniel J.R., Zolper J.C. The future of silicon on insulator (SOI) technology in microelectronic systems. Proceedings of the IEEE International SOI Conference.

[80] Maszara W.P., Lin M.R. FinFET-Technology and circuit design challenges. Proceedings of the IEEE ESSCIRC Conference.

[81] International Roadmap for Device and Systems (IRDS) 2021. https://irds.ieee.org/editions/2021.

[82] Ishibashi K., Osada K. (2011). Low Power and Reliable SRAM Memory Cell and Array Design.

[83] Pal S., Gupta V., Ki W.H., Islam A. (2019). Transmission Gate Based 9T SRAM Cell for variation resilient low power and reliable internet of things operation. IET Circuits Devices Syst..

[84] He Y., Zhang J., Wu X., Si X., Zhen S., Zhang B. (2019). A Half Select Disturb Free 11T SRAM Cell With Built in read/write Assist Scheme for Ultra Low Voltage Operation. IEEE Trans. Very Large Scale Integr. Syst. VLSI.

[85] Ichihashi M., Woo Y., Parihar S. SRAM cell Performance analysis beyond 10nm FinFET Technology. Proceedings of the IEEE International Symposium on VLSI Technology Systems and Applications (VLSI-TSA).

[86] Collinge J.P. (2008). FinFETS and Other Multi Gate Transistors.

[87] Chang W.T. (2021). Modifying Threshold Voltages to n and p type FinFETs by Work Functions Metal Stacks. IEEE Open J. Nanotechnol..

